# ﻿Four new species of the lichen genus *Diorygma* (Graphidaceae, Ostropales) from Guizhou, China

**DOI:** 10.3897/mycokeys.121.157714

**Published:** 2025-08-22

**Authors:** Wei Wu, Shu-Hao Jiang, Lin-Shan Chai, He-Yun Bo, Ruvishika S. Jayawardena, Shao-Bin Fu, Qing-Feng Meng

**Affiliations:** 1 School of Pharmacy, Zunyi Medical University, Zunyi, Guizhou Province 563000, China; 2 College of Agriculture and Biology, Liaocheng University, Liaocheng, Shandong Province 252059, China; 3 Center of Excellence in Fungal Research, Mae Fah Luang University, Chiang Rai 57100, Thailand; 4 School of Science, Mae Fah Luang University, Chiang Rai 57100, Thailand; 5 School of Public Health, Zunyi Medical University, Zunyi, Guizhou Province 563000, China

**Keywords:** 4 new species, lichenized fungi, morphology, muriform, phylogeny

## Abstract

Four new species of the lichen genus *Diorygma* from China are described based on morphological, chemical, and phylogenetic evidence. Phylogenetic analyses were conducted using both RAxML and Bayesian posterior probability inference, based on combined LSU and mtSSU sequences. The characteristic compounds were analyzed by colorimetric reactions and thin-layer chromatography. *Diorygma guizhouense* is characterized by small apothecia, a narrow to slightly open disc covered with white pruina, and the presence of stictic, salazinic, and norstictic acids. *Diorygma leigongshanense* is characterized by small, oval apothecia, a disc surrounded by entire, raised, and widely open thalline margins covered with thin, pale yellowish pruina, and the same chemical substances as *D. guizhouense*. *Diorygma locitonitrus* is distinguished by the presence of salazinic acid and hyaline, richly muriform ascospores, notably with distinctly smaller peripheral cells compared to the central cells. *Diorygma weii* is characterized by stellately branched apothecia with a closed to slit-like disc, a milky white thallus tinged with green, and the presence of only norstictic acid. Detailed morphological descriptions and illustrations of the new species are provided, along with a comprehensive species checklist highlighting the diagnostic characteristics of the known species in this genus.

## ﻿Introduction

*Diorygma* was described by Eschweiler (1824), who mentioned *Opegrapha hieroglyphica* as a member of this genus but did not formally designate it as the type species. Later, Staiger (2002) synonymized *Opegrapha hieroglyphica* as *Diorygma hieroglyphicum* and designated it as the lectotype for *Diorygma*. The genus is widely distributed, primarily in tropical regions, and often thrives on the sheltered or overhanging sides of trees (Staiger 2002; Kalb et al. 2004). It is characterized by a crustose, white to pale olive-green thallus and lirelliform to irregularly rounded ascomata with a pruinose disc. The exciple is uncarbonized or slightly carbonized, and the hymenium is hyaline, not inspersed, with branched or anastomosing paraphyses that have a thick gelatinous wall. The asci are clavate and contain 1–8 spores. The ascospores are hyaline (rarely brownish), transversely septate to mostly muriform (Li and Jia 2016). Chemically, the genus produces compounds such as norstictic acid, stictic acid, or the protocetraric acid complex (Kalb et al. 2004; Aptroot et al. 2023).

Kalb et al. (2004) provided a monograph on the genus *Diorygma*, which included 24 recognized species, a detailed taxonomic key, and a phylogenetic tree based on LSU sequences. Subsequently, Sharma and Makhija (2009a, 2009b) described four additional species characterized by the presence of norstictic and salazinic acids as the major secondary metabolites. Later, Sharma and Khadilkar (2012) reported four more new species from India, two of which exclusively contain norstictic and salazinic acids. Feuerstein et al. (2014) expanded the diversity of the genus by describing three new species and providing a revised global key encompassing 52 species. Numerous additional taxa have been discovered and reported, further increasing diversity (Cáceres 2007; Makhija et al. 2009; Sharma and Makhija 2009a; Lima et al. 2013; Feuerstein et al. 2014; Sutjaritturakan et al. 2014; Aptroot et al. 2023, 2024). According to the latest phylogenetic research by Ansil et al. (2023), *D. dandeliense* B.O. Sharma & Khadilkar is a synonym of *D. karnatakense* B.O. Sharma & Khadilkar. Currently, 89 species of the genus *Diorygma* are recognized worldwide (Table 2). Molecular data for 15 known species and 4 novel species are available (Table 1).

**Table 1.** Taxon name, voucher/culture, and GenBank accession numbers used in this study. Newly generated sequences are shown in bold, and “^T^” indicates holotype strains. Absence of GenBank accession numbers is indicated by “NA”.

Taxon	Locality	Voucher/Strain	GenBank accessions number	
			mtSSU	LSU
*Diorygma antillarum*	Brazil	Nelsen 4185 (F)	JX046451	JX046464
*D. antillarum*	USA	Nelsen 4037 (F)	JX046452	JX046465
*D. antillarum*	El Salvador	Lücking 33018 (F)	JX046453	NA
*D. antillarum*	El Salvador	Lücking 33019 (F)	JX046454	JX046467
*D. circumfusu*	Australia	Kalb 33922 (Herb. Kalb)	DQ431963	NA
*D. defectoisidiatum*	Brazil	Cáceres & Aptroot 28966a^T^ (ISE)	OR270821	NA
*D. defectoisidiatum*	Brazil	Cáceres & Aptroot 28966b (ISE)	OR270822	NA
***D. guizhouense***	**China**	**L 0093724^T^ (KUN)**	**PQ691396**	**PQ691395**
*D. hieroglyphicum*	French	Wirth 26647	NA	AY640015
*D. junghuhnii*	Australia	Kalb 33937 (Herb. Kalb)	DQ431962	NA
*D. junghuhnii*	Fiji	Lumbsch 20539l (F)	JX421023	JX421474
*D. junghuhnii*	Australia	Kalb 33931 (Herb. Kalb)	NA	AY640017
*D. junghuhni*	Brazil	Kalb 33254 (Herb. Kalb)	NA	AY640016
*D. junghuhnii*	India	Mycobiont (MSSRF)	MN944821	NA
*D. junghuhnii*	India	Thallus (MSSRF)	MN944822	NA
*D. karnatakense*	India	21.26 (AMH)	OP235521	OP235516
*D. karnatakense*	India	21.52 (AMH)	OP235522	OP235517
*D. karnatakense*	India	21.54 (AMH)	OP235523	OP235518
*D. karnatakense*	India	21.55 (AMH)	OP235524	OP235519
*D. karnatakense*	India	21.60 (AMH)	OP235525	OP235520
***D. leigongshanense***	**China**	**L 0093725^T^ (KUN)**	**PQ847480**	**NA**
***D. locitonitrus***	**China**	**L 0093723^T^ (KUN)**	**PQ847478**	**PQ847477**
*D. microsporum*	USA	Lücking 26504 (F)	JX421024	NA
*D. minisporum*	Kenya	Lumbsch 19543v (F)	HQ639598	HQ639626
*D. minisporum*	USA	Lücking 26564 (F)	NA	HQ639665
D. aff. minisporum	South Africa	Medeiros 2106 (DUKE)	ON507279	ON507251
*D. tibellii*	Nicaragua	Lücking 28533 (F)	JX421025	JX421475
*D. poitaei*	Nicaragua	Lücking 28538 (F)	HQ639596	HQ639627
*D. pruinosum*	Australia	Mangold 28g (F)	NA	JX421476
*D. pruinosum*	Australia	Kalb 26578 (Herb. Kalb)	NA	AY640014
*D. sipmanii*	Costa Rica	Lücking 14011 (F)	DQ431961	AY640020
*D. tiantaiense*	China	Jia ZJ19123^T^ (LCUF)	NA	MW750692
*D. toensbergianum*	Brazil	Cáceres & Aptroot 42003^T^ (ISE)	OR270820	NA
*Diorygma* sp.	Australia	Lumbsch 19082l (F)	NA	JX421479
*Diorygma* sp.	Fiji	Lumbsch 20501l (F)	NA	JX421478
*Diorygma* sp.	Fiji	Lumbsch 20513a (F)	NA	JX421477
***D. weii***	**China**	**L 0093727^T^ (KUN)**	**PQ847479**	**NA**
*Glyphis cicatricosa*	El Salvador	Lücking 28047 (F)	HQ639610	JX421505
*G. cicatricosa*	Kenya	Lumbsch 19528o (F)	JX421062	JX421503

**Table 2.** Morphological comparison of *Diorygma* species.

	Species name	Ascomata	Disc	Exciple	Hymenium	Spores num.	Size/μm	Septa	Chemistry	References	Notes
1	*Diorygma aeolum* (Stirt.) Pushpi Singh & Kr.P. Singh		disc closed to slit-like	exciple uncarbonized, convergent to slightly divergent	I+ violet	4–6, I+ violet	(76–) 90–120 × 26–40	muriform, all locules of equal size	stictic acid (major), constictic acid (minor)	Singh and Singh (2017)	
2	*Diorygma africanum* Kalb, Staiger & Elix	1–5 × 0.5–1 mm, often separated by a deep slit	disc open	exciple divergent, brownish-carbonised	I– or I+ weakly bluish	1, I– or I+ weakly bluish-violet	140–200 × 40–65 μm	densely muriform, peripheral < central	protocetraric acid; type collection with additional 4–0–methylhypoprotocetraric acid	Kalb et al. (2004)	immersed, bacilliform conidia, 4.5 × 1 μm, acrogenously
3	*Diorygma agumbense* B.O. Sharma & Khadilkar	1–1.5 mm	disc narrow, slit-like, rarely ± open	exciple convergent	I+ blue	8 arranged in rows, I+ blue violet	87–125 × 20–37 µm	muriform, peripheral = central	cryptostictic, protocetraric, and stictic acids present (K+ yellow, P+ red)	Sharma and Khadilkar (2012)	
4	*Diorygma alagoense* Cáceres & Lücking	aggregate, 0.5–1.5 × 0.4–0.7 mm.	disc concave to plane		colorless	6–8, I+ violet-blue	40–60 × 10–14 μm	muriform	lichexanthone (thallus only, UV+ bright yellow), stictic acid	Cáceres (2007)	
5	*Diorygma albocinerascens* Makhija, Chitale & B.O. Sharma	0.5–6 × 0.2–1 mm, 2–3-striate	disc narrow, slit-like	exciple convergent	I+ blue	1–2, I+ violet	135–150 × 18–27 µm	muriform, peripheral = central	cryptostictic, methylstictic, norstictic, and salazinic acids (major)	Makhija et al. (2009)	
6	*Diorygma albovirescens* Makhija, Chitale & B.O. Sharma	0.5–3 × 0.2–0.5 mm, fissure like	disc narrow, slit-like		I+ blue	4–8, I+ violet	66–99 × 12–36 µm	muriform, peripheral = central	constictic, cryptostictic, and stictic acids (major)	Makhija et al. (2009)	
7	*Diorygma album* (Müll. Arg.) Lücking, M. Cáceres & Aptroot	perithecium basally incomplete, thin				1	90 × 33 μm	transverse, containing 5–7 locelli		Aptroot et al. (2024)	= *Graphina alba* Müll.Arg.
8	*Diorygma angusticarpum* J. Sutjaritturakan & K. Kalb	0.2–1.5 × 0.1–0.2 mm	disc slit like when young, becoming ± open when old, rarely widely opened		I+ blue	1, I+ violet	90–110 × 30–37 µm	richly muriform, 20–28/8–10 locular peripheral = central	stictic and salazinic acids (major), cryptostictic acid (minor), consalazinic acid (submajor), norstictic acid and hypostictic acid (trace)	Sutjaritturakan et al. (2014)	
9	*Diorygma antillarum* (Vain.) Nelsen, Lücking & Rivas Plata	pseudisidia numerous, cylindrical, unbranched or sparsely branched				asci and pycnidia not seen			salazinic and norstictic acids [major], with or without protocetraric acid, rarely additionally with atranorin and zeorin detected with TLC; thallus and prothallus C–, K+ yellow then red, P+ orange, UV–, I– and K/I–	Aptroot et al. (2009); Matthew et al. (2012)	= *Chiodecton antillarum* Vain. = *Herpothallon antillarum* (Vain.) Aptroot, Lücking & G. Thor
10	*Diorygma archeri* S. Joshi & Hur	2.0–3.5 × 0.55–0.93 mm	disc initially closed, later exposed	exciple poorly developed, entire, non-carbonized, hyaline to pale brown, 20–30 μm thick	I+ blue or weakly blue	1, I+ violet-blue	160–255 × 56–87 µm	muriform, peripheral = central	thallus K– or K+ slightly yellow, PD+ yellow-orange, C–, KC–, TLC: protocetraric acid	Joshi et al. (2013)	
11	*Diorygma australasicum* (Elix) Lücking, Elix & A.W. Archer	1–6 mm × 0.4–0.5 mm, 0.25–0.35 mm high				1	70–110 × 20–25 µm	richly muriform	norstictic acid (major), connorstictic acid (minor)	Archer and Elix (2009)	possession isidia
12	*Diorygma basinigrum* F. Seavey & J. Seavey	1–3 × 0.15–0.30 mm	disc closed or slightly open	exciple carbonized basally, lower lateral part occasionally also thinly carbonized	I–	8, oblong, I+ dark purple	24–32 × 5.5–6.5 µm	8–10-celled	K+ yellow, KC–, C–, P+ orange. TLC: stictic acid	Seavey and Seavey (2014)	
13	*Diorygma biforme* Eschw.	apothecia precisely linear, much longer and narrower, with branches and sub-branches that are alternate, bifid, dense, and slightly interwoven. Margins parallel, with apices that are pointed or rounded and sometimes club-shaped								Eschweiler (1833)	
14	*Diorygma cameroonense* Kalb	0.5–2 × 0.1–0.2 mm	disc initially concealed, wide open with age	exciple entire, beige, 15–30 µm thick, laterally covered by algiferous thallus, without or very few crystals	I–	8, with thickened septa and lentiform lumina, I+ strongly violet-blue	(10–)15–20 × 5–8 µm	submuriform with 3–5 transverse and 0–1 longitudinal septum in 1 or 2 (very seldom 3)	stictic acid (major), menegazziaic acid (minor), constictic acid (submajor)	Kalb (2020)	
15	*Diorygma chumphonense* J. Sutjaritturakan & K. Kalb	1–3 × 0.5–0.9 mm	disc widely open	exciple divergent,not well developed, basally orange brown	I–	1, ends with gelatinous caps, I+ violet	95–110 × 37–40 µm	richly muriform, peripheral < central, 19–20 × 7–10 locular	salazinic acid (major) and norstictic acid (minor)	Sutjaritturakan et al. (2014)	
16	*Diorygma circumfusum* (Stirt.) Kalb, Staiger & Elix	numerous, long, ± flexuous and branched, 0.5–4 × 0.2–0.4 mm	disc open	exciple divergent, uncarbonised, poorly developed	I+weakly bluish violet	(2–)4–8, one tapering end, young spores often with gelatinous caps, I+ violet	60–100 × 8.5–16 μm	transversely septate, 13–23 locular, with lentiform lumina	norstictic acid (major), cryptostictic acid (minor), connorstictic acid (trace), stictic acid (usually trace)	Kalb et al. (2004)	
17	*Diorygma citri* J. Sutjaritturakan & K. Kalb	lirellate, numerous, short to long, ± flexuous and branched, 0.2–1.0 × 0.1–0.3 mm	disc slightly to widely opened, with a powdery reddish brown pruina		I–	1, without gelatineous sheath, I+ violet	60–70 × 20–30 µm	richly muriform, 14–17/4–10 locular, peripheral = central	salazinic acid (major), hypostictic acid (major), methylsticic acid (minor) and two unknown substances	Sutjaritturakan et al. (2014)	
18	*Diorygma confluens* (Fée) Kalb, Staiger & Elix	numerous, short or long, ± flexuous and branched, partly lobed, 0.3–5 × 0.2–0.8 mm, bulging thalline margins separated by a deep slit	disc open	exciple divergent, basally and laterally well developed, with a very variable carbonisation	I+ weakly violet	1, with a thin spore wall, I+ violet	(80–)95–135(–145) × 25–45(–50) μm	muriform, peripheral ≤ central, 20–36/5–12 locular	Iichexanthone and stictic and constictic acids (major), cryptostictic, hypoconstictic substictic and hypostictic acids (trace or absent)	Kalb et al. (2004)	pycnidia in small warts, with dark brown ostiolum, and a yellowish wall; conidia acrogenously (conidiophore-typeI), hyaline, bacilliform, c.4–5 × 1 μm
19	*Diorygma conprotocetraricum* J. Sutjaritturakan & K. Kalb	ascomata lirellate, numerous, simple to branched, 0.4–2.0 × 0.2–0.5 mm	disc open, slightly opened when young, with a granular whitish pruina	exciple divergent, some parts of bark rise and form a layer parallel to the exiple, orange brown	I+ blue	1, without gelatinous sheath, I+ violet	50–96 × 20–36 µm	richly muriform, peripheral = central, 19–28/5–8 locular	conprotocetraric acid (major), stictic acid (minor) and cryptostictic acid (minor)	Sutjaritturakan et al. (2014)	
20	*Diorygma dandeliense* B.O. Sharma & Khadilkar	0.5–2 mm long	disc narrow, slit-like, rarely ± open	exciple convergent, non-carbonized, non-striate	I+ blue	1, I+ blue violet	125–175 × 27–35 µm	muriform, peripheral = central, regularly arranged in rows	norstictic and salazinic acids present (K+ yellow forming red crystals)	Sharma and Khadilkar (2012)	= *Diorygma*karnatakense B. O. Sharma & Khadilkar
21	*Diorygma dealbatum* B.O. Sharma & Makhija	0.5–2.5 mm long	disc narrow to broad	exciple divergent, base distinctly orange brown	I+ blue	1, I+ blue	105–147 × 33–37 μm	muriform, peripheral = central	norstictic and salazinic acids present (K+ yellow forming red crystals)	Sharma and Makhija (2009a)	
22	*Diorygma defectoisidiatum* Aptroot & M. Cáceres	ascomata and pycnidia not observed				not observed			thallus UV–, K–, P–. TLC: nil	Aptroot et al. (2023)	having isidia
23	*Diorygma epiglaucum* (Müll. Arg.) Kalb, Staiger & Elix	1–6 × 0.2–0.5 mm, lirellae fissurine, bulging thalline margins, separated by a deep slit	disc open	exciple diver-gent, basally and laterally well developed, with avery variable carbonisation	I– or I+ weakly violet	1, I+ violet or I-or only the very thin endospore I+ blue violett	(120–)135–203 × 35–70 μm	muriform, peripheral < central	lichexanthone and stictic acid(major), constictic, cryptostictic, hypoconstictic, sub.stictic, and hypostictic acids (trace or absent)	Kalb et al. (2004)	
24	*Diorygma erythrellum* (Mont. & Bosch) Kalb, Staiger & Elix	often numerous, short, oval to oblong, ± flexuous and branched, 0.7–7 × 0.3–0.5 mm; lirellae fissurine	disc ± open	exciple divergent,completely uncarbonised, yellowish to pale brown	I+ blue	8, with halo when young, I+ violet	30–65 × 12–20 μm	muriform, peripheral = central, 9–15/2–4 locular	norstictic acid (major), connorsticticacid (minor or trace), stictic acid (trace or absent)	Kalb et al. (2004)	pycnidia immersed in slightly elevated thalline warts with pale yellowish ostiola; conidia hyaline, bacilliform, 3.5–4 × 1–1.3 μm
25	*Diorygma excipuloconvergentum* Makhija, Chitale & B.O. Sharma	1–6 mm long and 0.1–0.25 mm	disc white, narrow, slitlike, later moderately open	exciple poorly developed, pale yellow, indistinctly 2–4-striate, blackish brown at apices, present at the base, converging at the apical portion	I+ blue	1–2(–4), I+ violet	147–273 × 34–67 µm	muriform, peripheral = central	constictic, cryptostictic, methylstictic, norstictic, and salazinic acids (major)	Makhija et al. (2009)	
26	*Diorygma extensum* H. J. M. Sipman	0.2–0.3 mm wide, up to 1 mm long	closed		I–	8, bacillar, I+ weakly blue-violet	18–20 × 5–6 µm	transversely 4–6-septate, with lenticular lumina	norstictic acid	Sipman (2014)	
27	*Diorygma fissuroxanthonicum* Aptroot & Schumm	immersed, lirelline, wavy usually branched, up to 2 mm long and 0.3 mm wide	disc slit-like, pinkish		I+ violet	1, I+ violet	45–55 × 15–17 µm	muriform, 9–11 × 2–5-septate	UV+ yellow, lichexanthone	Müller (1895); Schumm and Aptroot (2024){Schumm, 2024 #59}{Schumm, 2024 #59}	
28	*Diorygma fuscopruinosum* J. Sutjaritturakan & K. Kalb	ascomata lirellate, short to long, ± flexuous and 1–branched, 0.5–3 × 0.5–1 mm	disc widely open, brown with a granular brownish pruina	exciple divergent, laterally with parts of the bark, dark brown	I+ dark blue	1, without gelatinous sheath, I+ violet	60–70 × 20–35 µm	richly muriform, peripheral = central, 17–20/3–10 locular	stictic acid (major) and constictic acid (minor)	Sutjaritturakan et al. (2014)	
29	*Diorygma fuscum* Jian Li bis & Z.F. Jia	1–4 × 0.3–2 mm	disc open	exciple divergent, laterally uncarbonized, rudimentarily developed, consisting of a weakly and irregularly or brownish hyphal tissue intermingled with parts of the substrate, carbonization sometimes restricted to the basal position	I+ weakly bluish violet	8, with thin halo, I+ violet	40–60 × 12–18 µm	muriform, peripheral = central, 10–14/3–4-locular	stictic acid (major), constictic, hypostictic and hypoconstictic acids (minor, trace or absent)	Li and Jia (2016)	
30	*Diorygma guizhouense* Wei Wu & S.B. Fu, sp. nov.	erumpent, simple or irregularly branched, 2–5 mm long, and 0.2–0.4 mm wide	disc narrow to slightly open, covered with a white pruina	exciple uncarbonized, brown at apex, pale yellowish brown towards base	I+ weakly blue-violet	1, I–	(104–)125–119(–214) × (30–)36–58(–77) μm	muriform, 24–32 × 5–12 locular, peripheral = central	thallus K+ reddish brown, C–, KC+ orange, P+ yellow, TLC: stictic acid, salazinic acid, norstictic acid.	this study	
31	*Diorygma gyrosum* Aptroot, Lücking & M. Cáceres	solitary, rounded, aggregated in groups of 3–7 × 0 .5–0.9 mm wide	disc closed			2, without gelatinous sheath, I+ violet	70–80 × 20–24 μm	muriform	thallus UV+ yellow, K+ red, P–. TLC: lichexanthone and salazinic acid	Aptroot et al. (2023)	
32	*Diorygma hieroglyphicellum* J. Sutjaritturakan & K. Kalb	1.5–15 × 1–2 mm; thalline margins thick, bulging	disc first slit-like, later widely opened	exciple divergent, brown to orange-brown, very thin	I+ very weakly blue	1, I+ violet	75–100 × 20–32 μm	richly muriform, peripheral = central, 15–21/4–6 locular	stictic acid (major), norstictic acid (trace) and an unknown substance	Sutjaritturakan et al. (2014)	
33	*Diorygma hieroglyphicum* (Pers.) Staiger & Kalb	numerous,short or oblong, ± fexuous and branched, 0.4–3 × 0.2–0.5 mm, separated by a deep slit	disc narrow to slightly open	exciple divergent, poorly developed, with a very variable carbonisation	I+ violet	1, I+ violet	95–150(–170) × 30–45 µm	muriform, peripheral = central, 23–32/6–9 locular	stictic and norstictic acids (major, butsee under Remarks), cryptostictic, substictic, 3-0methylconsalazinic, constictic, hypostictic andconnorstictic acids (trace or absent)	Staiger (2002); Kalb et al. (2004)	
34	*Diorygma hololeucum* (Mont.) Kalb, Staiger & Elix	often numerous, long, ± flexuous and branched, 1–10 × 0.6–1 mm; lirellae large	disc open	exciple divergent, totally uncarbonised	I+ bluish violet	(1–)2–4(–6or–8)/ascus, hyaline, ends with gelatinous caps, I+ violet	125–230(–250) × 30–45(–50) μm	densely muriform, peripheral and central spore locules of ± equal size, 28–40/5–8 locular	protocetraricacid. One collection contained additional conprotoce- traric acid (submajor) and subvirensic acid (trace)	Kalb et al. (2004)	
35	*Diorygma hydei* Meng & Wei Wu	1–4 × 0.5–2 mm, lirellate, oblong to elongated, ± flexuous, either simple or branched	disc irregularly wide open, covered with a white pruina, and surrounded by entire, raised, swollen margins	exciple uncarbonized, not convergent, brownish both basally and laterally	non-inspersed, I+ violet	1–2, I+ violet	(86–)150–202(–238) × (32–)36–47(–50) µm	muriform, peripheral = central	thallus K+ reddish brown, C–, KC+ yellow, P+ yellow, TLC: constictic acid, salazinic acid, norstictic acid	Hongsanan S et al. (2025)	
36	*Diorygma inaequale* B.O. Sharma & Makhija	1–6 mm long	disc more or less open	exciple non striate, divergent, non-carbonized, distinctly orange at the base	I+ blue	1, I+ blue violet	79–96 × 29.4–33.6 µm	muriform, peripheral = central	norstictic and salazinic acids present	Sharma and Makhija (2009a)	
37	*Diorygma incantatum* S.C. Feuerst. & Eliasaro	circular to elongated, simple, with rounded ends, 0.6–1.6 × 0.3–0.6 mm	disc exposed		I–	8, filiform, surrounded by gelatinous sheath, I–	105–108 × 6 µm	transversely 29–31-septate	K+ pale yellow. TLC: an unidentified compound forming a spot of purple colour, in UV fluorescent orange, at approximately Rf 44 in solvent system C	Feuerstein et al. (2014)	
38	*Diorygma inexpectatum* J. Sutjaritturakan & K. Kalb	simple or with a few branches, 0.3–2.5 × 0.1–0.3 mm	disc slit-like to widely open	exciple divergent, totally uncarbonised, poorly developed	I+ dark blue	1, I+ violet	65–90 × 25–35 µm	richly muriform, peripheral = central, 15–20/7–9 locular	salazinic acid (major) and hypostictic acid (minor)	Sutjaritturakan et al. (2014)	
39	*Diorygma intermedium* Kalb, Staiger & Elix	0.5–4 × 0.2–0.6 mm; lirellae appearing as fissures	disc narrow rarely ± open	exciple slightly convergent, uncarbonised	I+bluish violet	(4–)6–8, with thin halo, I+ violet	(60–) 70–115 × 16–22 μm	muriform, peripheral = central, 18–27/3–5locular	hyposticticand hypoconstictic acids (major), a-acetylhypoconstictic acid (minor, trace or absent)	Kalb et al. (2004)	
40	*Diorygma isabellinum* (Zahlbr.) Z.F. Jia & Lücking	2–4.5 × 0.2–0.35 mm	discs closed to slightly opened	exciple not carbonized	I–	1, I+ violet	110–120 × 35–48 µm	muriform	norstictic acid (major), connorstictic acid (minor or trace)	Jia and Lücking (2017)	
41	*Diorygma isidiatum* Swarnal.	0.8–6 × 0.2–0.6 mm	disc concealed to slightly open	excipulum complete, uncarbonized	I+ blue-violet at lateral and the apical parts	asci without ascospores.			thallus K+ dark red, C–, P+ yellow; UV–; salazinic acid (major) detected by TLC	Swarnalatha (2021)	isidia numerous
42	*Diorygma isidiolichexanthonicum* Aptroot	isidia numerous, covering most of the central part of the thallus, cylindrical, simple to irregularly branched, 0.1–0.7 mm high, c. 0.1 mm wide				ascomata and pycnidia not observed			thallus and apothecia UV+ yellow, C–, P–, K–. TLC: lichexanthone	Aptroot and Feuerstein (2020)	isidia numerous
43	*Diorygma junghuhnii* (Mont. & Bosch) Kalb, Staiger & Elix	roundish, oval to distinctly elongated, 1–5 × 0.2–0.5 mm	disc wide	exciple slightly divergent, uncarbonised, poorly developed	I+ distinctly bluish violet	1 (rarely2), I+ violet	(60–)80–125 × 21–42 μm	muriform, peripheral = central, 25–31/6–10 locular	norstictic acid (major), connorstictic acid (minor)	Kalb et al. (2004)	
44	*Diorygma karnatakense* B.O. Sharma & Khadilkar	straight to curved, immersed to erumpent, edges acute, 0.2–5 mm long, 0.2–0.4 mm wide, same level to slightly raised	disc concealed, brownish black, with white pruina	exciple reduced, entire, convergent,non-carbonized, brown at apex, pale yellowish brown towards base	I+ blue	1–8, I+ blue-violet	75–220 × 18.5–51.5 μm	muriform, peripheral = centra	thallus and ascoma UV–, K+ yellow with crystals. TLC: norstictic and salazinic acids	Ansil et al. (2023)	
45	*Diorygma kurnoolensis* Mohabe, Nayaka & A.M. Reddy	rare, simple to branched, 0.2–0.7 mm long	disc concealed to slightly open	exciple non-carbonized, brownish, well developed, labia convergent to rarely divergent	slightly I+ blue laterally	1–2, ellipsoid to fusiform, I+ slightly blue-violet or pale orange	50–105 × 15–25 μm	muriform, 14–18/3–8 cells, peripheral = central	thallus and medulla K+ deep yellow, C–, KC–, P–, TLC: stictic (major), cryptostictic and constictic acid (trace) present	Mohabe et al. (2015)	
46	*Diorygma leigongshanense* Wei Wu & S.B. Fu, sp. nov.	± flexuous, simple or with a few branches, 0.7–2.5 mm long, and 0.4–1.2 mm wide	disc surrounded by entire raised thalline margins, widely open, covered with a thin, pale yellowish pruina	exciple uncarbonized, basally and laterally brownish	I–	1, I–	(95–)119–170(–194) × (22–)27–46(–54) μm	muriform, 20–42 × 5–10 locular, peripheral = central	thallus K+ reddish brown, C–, KC+ yellow, P+ yellow, TLC: stictic acid, salazinic acid, norstictic acid	this study	
47	*Diorygma locitonitrus* Wei Wu & S.B. Fu, sp. nov.	erumpent, simple or irregularly branched, 0.5–2.5 mm long, and 0.2–0.6 mm wide	disc narrow to open, covered with a white pruina	exciple uncarbonized, brown at apex, pale yellowish brown towards base	I+ weakly blue-violet	1, I–	(105–)117–189(–247) × (25–)33–60(–76) μm	muriform, 26–40/6–15 locular, peripheral < central	thallus K+ reddish brown, C–, KC+ orange, P+ yellow, TLC: constictic acid, salazinic acid, norstictic acid	this study	
48	*Diorygma lichexanthonicum* Aptroot, Lücking & M. Cáceres	solitary, linear, not branched but wavy, 3 × 0.4–0.7 mm	disc flat, open, ~0.3 mm wide	labia cracked or crenulated	inspersed	4, without gelatinous sheath, I+ violet	60–67 × 16–18 µm	muriform	thallus UV+ yellow, K–, P–. TLC: lichexanthone	Aptroot et al. (2023)	
49	*Diorygma longilirellatum* B.O. Sharma & Makhija	up to 9 mm long	disc slightly open	exciple convergent to divergent	I+ blue	1, I+ violet	105–113 × 33–42 µm	muriform, peripheral = central	norstictic acid present	Sharma and Makhija (2009b)	
50	*Diorygma longisporum* M. Cáceres & Aptroot	linear and usually branched, 0.2–0.4 mm wide, 1–4 mm long	disc exposed			8, long cylindrical, ends with small gelatinous caps	100–115 × 15–18 μm	20–30/0–1-septate	thallus and ascomata UV–, C–, P–, K+ yellow red. TLC: norstictic acid	de Lima et al. (2013)	
51	*Diorygma macgregorii* (Vain.) Kalb, Staiger & Elix	1–5 × 0.4–2 mm	discs wide	exciple divergent	I+ weakly bluish violet	1, I+ violet (a few also I–)	135–180 × 40–63 µm	muriform, peripheral < central	norstictic acid (major), connorsticticacid (minor); cryptostictic acid (major) and sticticacid (minor) are additionally found in the type specimen	Kalb et al. (2004)	
52	*Diorygma manipurense* B.O. Sharma & Makhija	0.2–0.3 × 0.4–0.5 mm	disc narrow to distinctly open	exciple convergent to divergent, with 2–5 thin striae, apically dark brown, colourless at the base	I+ blue	2–4, I+blue violet	76–92 × 21–25 µm	muriform, peripheral = central	norstictic and salazinic acids present.	Sharma and Makhija (2009a)	
53	*Diorygma megaspermum* Makhija, Chitale & B.O. Sharma	ascocarps lirelline, 0.5–9 mm long and 0.1–0.25 mm broad	disc narrow	exciple poorly developed, convergent, 2–6striate, blackish brown at apices and dark brown laterally, present at base	KI+ blue;	1, without gelatinous sheath,I+ violet	231–244 × 59–76 µm	muriform, peripheral = central	consalazinic, constictic, cryptostictic, and stictic acids (major)	Makhija et al. (2009)	
54	*Diorygma megasporum* Kalb, Staiger & Elix	numerous, round, oval to oblong partly in rows, 0.5–4 × 0.2–0.7 mm; lirellae immersed, partly separated by a slit	disc narrow to slightly open	exciple slightly convergent, yellowish	I+ blue-violet	2–6, the spores have quite different sizes, partly with 2–3 μm thick gelatinous wall, I– or I+ violet	80–170 (–220) × 21–55 μm	muriform, peripheral = central, 28–57/7–12 locular	stictic, a-acetylconstictic and constictic acids (major), norstictic and hypostictic acids (trace)	Kalb et al. (2004)	
55	*Diorygma megistosporum* Makhija, Chitale & B.O. Sharma	ascocarps lirelline, 1–4 mm long and 0.1–0.3 mm broad	disc narrow, concealed, slit like or broader, blackish brown to black, white pruinose when open	poorly developed, 3–4 indistinct striation, blackish brown at apices, present at the base, pale yellow to almost hyaline, apically convergent, covered by a thick thalline margin up to the top	I+ blue	1–4, fusiform-oblong, without gelatinous sheath, I+ violet	151–294 × 38–63(–84) µm	muriform, peripheral = central	cryptostictic, norstictic, and stictic acids present	Makhija et al. (2009)	
56	*Diorygma microsporum* M. Cáceres & Lücking	1–5 mm long, 0.15–0.25 mm wide	disc partly exposed			8, strongly I+ violet-blue	12–15 × 6–7 μm	submuriform with 3 transverse and 0–1 longitudinal septum	norstictic acid	Lumbsch et al. (2011)	
57	*Diorygma minisporum* Kalb, Staiger & Elix	elongate, ± fexuous and branched, 2–5 × 0.1 mm; lirellae fissurine, separated by a slit	disc narrow, slightly open	exciple divergent, uncarbonized, laterally poorly developed, excipular base well developed	I+ weakly blue	8, I+ violet	17–20 × 5–6.5 μm	transversely septate,6–8 locular	hypostictic, hypoconstictic, sticticand constictic acids (major)	Kalb et al. (2004)	
58	*Diorygma monophorum* (Nyl.) Kalb, Staiger & Elix	0.5–5 × 0.4–0.5 mm, often separated by a slit	disc open	exciple divergent or slightly convergent uncarbonised, basally poorly developed, laterally grey brown, partly striate by degenerated	I+ distinctly violet	1(–2), I+ violet	105–165 ×35–60 μm	muriform, peripheral < central	hypostictic, hypoconstictic and acetylhypoconstictic acids (maior or submaior)	Kalb et al. (2004)	pycnidia immersed in small thalline warts with a darker ostiolum: conidia hyaline. bacilliform, 3–4 × 1–1.5 μm
59	*Diorygma nigricans* Rivas Plata & Lücking	0.5–3 mm long, 0.2–0.4 mm wide	disc exposed, brown-black, non-pruinose			1, I+ violet-blue.	80–120 × 20–30 μm	richly muriform	lichexanthone (thallus surface UV+yellow), stictic and constictic acids (major), cryptostictic, hypostictic, and hypoconstictic acids (minor or traces)	Plata and Lücking (2013)	
60	*Diorygma norsubmuriforme* Aptroot, Lücking & M. Cáceres	solitary or in loose groups, linear, but repeatedly branched, ~0.2 mm wide, up to 5 mm in diam	disc flesh-colored, ~0.05 mm wide	excipulum completely uncarbonized.		8, without gelatinous sheath, I+ violet	6–7 × 3–5 μm	1–3-septate to submuriform with up to 6 locules	thallus UV–, K+ yellow > red, P–. TLC: norstictic acid	Aptroot et al. (2023)	
61	*Diorygma occultum* (Adaw. & Makhija) Pushpi Singh & Kr. P. Singh	ascomata lirellate, immersed, simple to branched	disc narrow to exposed	exciple uncarbonized, covered by lateral thalline margin	I–	6–8, I+ blue	18–28 × 7–8 μm	transversely 5–7-septate	lichexanthone, norstictic and constictic acids	Singh and Singh (2020)	
62	*Diorygma panchganiense* Makhija, Chitale & B.O. Sharma	1–2 mm long and 0.5–1.3 mm	disc narrow, when open pruinose	exciple poorly developed, 2–3 striate, present at the base, thin, pale brown laterally, blackish brown at apices, convergent	I+ blue	1–2, I+ violet	75–99 × 24–30 µm	muriform, peripheral = central	norstictic, salazinic, and methylstictic acids present	Makhija et al. (2009)	
63	*Diorygma pauciseptatum* S.C. Feuerst., I.P.R. Cunha, Aptroot & M. Cáceres	0. 3–1.1 × 0.1 mm	disc exposed		I–	8, surrounded by gelatinous sheath, I+ blue-violet	28–32 × 7 µm	transversely 7–9-septate	K+ yellow, releasing orange-red, needle-shaped crystals in microscopic section. TLC: norstictic and connorstictic acids	Feuerstein et al. (2014)	
64	*Diorygma poitaei* (Fée) Kalb, Staiger & Elix	0.5–4 × 0.2–0.4 mm, often separated by a deep slit	disc narrow, rarely ± open.	exciple slightly convergent,totally uncarbonised	I+ bluish violet	(4–)6–8, I+ violet	40-65 × 10–18 μm	muriform, peripheral = central, 12–17/3–4-locular	hypostictic and hypoconstictic acids(major), a-acetylhypoconstictic, constictic and stictic acids (minor, trace or absent)	Kalb et al. (2004)	
65	*Diorygma pruinosum* (Eschw.) Kalb, Staiger & Elix	1–3.5 × 0.3–1 mm; lirellae fissurine, discs are often separated by a deep slit	disc open, rarely ± convex	exciple divergent, irregularly carbonised or brownish	I+ weakly bluish violet	1(–2), I+ violet	95–150(–170) × (19–)25–50 μm	muriform, 25–35(–45)/6–10 locular, peripheral = centra	protocetraric acid	Kalb et al. (2004)	
66	*Diorygma reniforme* (Fée) Kalb, Staiger & Elix	oval or oblong, 0.5–4 × 0.3–1 mm; lirellae stout, sessile, partly with constricted bases	disc wide flat.	exciple distinctly divergent, basally and laterally brownish or carbonised	I+ weakly violet	1, I– or I+ violet	110–230 × 35–80 μm	muriform, peripheral < central	norstictic, protocetraric and salazinic acids (major), subnorstictic and consalazinic acids(trace). One collection contains lichexanthone, protocetraric and salazinic acids	Kalb et al. (2004)	
67	*Diorygma roseopruinatum* Papong, Lücking & Parnmen	stellately branched, 3–7 mm long, 0.3–0.4 mm broad, 0.15–0.2 mm high	disc narrow, brown, white-pruinose suffused with patches of pink-red pruina			1	60–100 × 20–30 µm	richly muriform, subdistoseptate with angular lumina	norstictic acid and unknown pink-red pigment on the ascomata; thallus P+ orangered, microscopic section with K+ yellow efflux forming red, needle-shaped crystals; pigment K+ purple	Papong et al. (2014)	
68	*Diorygma rufopruinosum* (A.W. Archer) Kalb, Staiger & Elix	roundish-oval or oblong, 1–4 × 0.4–0.8 mm; lirellae stout	disc wide or narrow, flat	exciple distinctly divergent, totally uncarbonised, rudimentary	I– or I+ weakly bluish	1, I+ blue violet	120–155(–215) × 30–45(–63) μm	muriform, peripheral < central	norstictic, protocetraric and salazinic acids (usually major, but salazinic acid lacking in the type specimen), subnorstictic and consalazinic acids (trace)	Kalb et al. (2004)	
69	*Diorygma rufosporum* (Patw. & C.R. Kulk.) B.O. Sharma & Makhija	1–3 mm long, emergent, branched	disc yellowish to brownish, flat, wide, pruinose	exciple divergent	I+ blue	1, with 5–7.5 µm thick sheath, I+ violet	(135–)168–205 × 54–96 µm	muriform, peripheral = central	constictic (major) and stictic (major) acids present	Sharma and Makhija (2009b)	
70	*Diorygma rupicola* B.O. Sharma & Makhija	0.2–0.5 mm long, simple, or irregularly branched	disc narrow, rarely ± open	exciple convergent, apically 1–2 striate, carbonized at the tips	I+ blue	1–2, I+blue violet	88–147 × 25–42 µm	muriform, peripheral = central, regularly arranged in rows	constictic, cryptostictic, and stictic (all major) and norstictic (minor) present (K+ yellow)	Sharma and Khadilkar (2012)	
71	*Diorygma salazinicum* J. Sutjaritturakan & K. Kalb	ascomata lirellate, numerous, short to long, ± flexuous and branched, size 0.3–3.7 × 0.3–1 mm	disc widely opened, with thick powdery white to pale brownish pruina	exiple divergent, light brown to orange-brown, very thin	I–	1, I+ violet	100–145 × 30–45 µm	richly muriform, peripheral = central, 25–28/5–10 locular	stictic acid (major), salazinic acid (submajor), cryptostictic acid (minor), peristictic acid (minor) and norstictic acid (trace)	Sutjaritturakan et al. (2014)	
72	*Diorygma salvadoriense* Kalb, Staiger & Elix	1–10 × 0.4–0.8 mm; constricted at the base	disc open	exciple divergent, orange-brown to carbonised at the base, apically, carbonisation variable	I– or I+ weakly violet	1, I– or I+ violet	150–200(–230) × 50–75 μm	muriform, peripheral < central	norstictic and salazinic acids	Kalb et al. (2004)	pycnidia numerous, immersed in small warts, pear-shaped, with distinct orange to brown ostiolum and thin orange wall; conidia formed acrogenous, hyaline, bacilliform, c.4–5 × 1–1.5 μm
73	*Diorygma salxanthonicum* Aptroot, Lücking & M. Cáceres	solitary, linear, not branched, 0.3–0.5 mm wide, up to 1 mm long	disc closed.			1, without gelatinous sheath, I+ violet	130–140 × 29–32 µm	muriform	thallus UV+ yellow, K+ red, P–. TLC: lichexanthone and salazinic acid	Aptroot et al. (2023)	
74	*Diorygma saxicola* B.O. Sharma & Makhija	ascocarps concolorous, numerous, curved, short, more or less flexuous, round to elongate, simple to branched, very closely arranged, immersed to slightly emergent	disc narrow to broad, 0.2–0.3 mm broad	exciple divergent, non-carbonized	I+ blue violet	1–6, I+ violet	143–172 × 29–34 μm	muriform, peripheral = central	stictic, constictic and norstictic (trace) acids present	Sharma and Makhija (2009b)	
75	*Diorygma sipmanii* Kalb, Staiger & Elix	short to elongate, 0.5–4 × 0.2–0.4 mm; lirellae immersed, often separated by a deep slit	disc often narrow to ± open	exciple convergent or slightly divergent, basally rudimentary or yellowish-orange to brown, laterally poorly developed	I+ blue violet	2–6, with a 5μm thick gelatinous epispore when young, I– or I+ rarely weakly reddish violet	45–100 × 14–30(–40) μm	muriform, peripheral = central, 14–30/5–8 locular	hypostictic and hypoconstictic acids(major), a-acetylhypoconstictic acid (minor or absent)	Kalb et al. (2004)	
76	*Diorygma soozanum* (Zahlbr.) M. Nakan. & Kashiw.	1–5 × 0.4–0.6 mm	disc first narrow, later on wide	exciple divergent, uncarbonised, poorly developed	I+ weakly bluish violet	1, I+ violet	110–140 × 35–45 µm	muriform, peripheral = central, 20–30/7–8- locular	K+ red, P+ yellow to red; norstictic and connorstictic acids	Kalb et al. (2004)	
77	*Diorygma sophianum* E.L. Lima & Lücking	irregularly branched, 1–5 mm long, 0.4–0.6 mm broad	disc concealed to partly exposed, rather narrow	excipulum laterally carbonized	colorless to yellowish	1–2, I+ violet-blue	120–140 × 35–45 µm	richly muriform	thallus in section with Kt yellow efflux, forming red, needle-shaped crystals, P+ orange, UV+ pale yellow; norstictic acid (major), connorstictic acid (minor or trace), lichexanthone (minor)	de Lima et al. (2019)	
78	*Diorygma spilotum* (Stirt.) Pushpi Singh & Kr.P. Singh	apothecia lirelliform, simple, immersed, brownish black	disc exposed	exciple uncarbonized, divergent	weak I+ blue	1, I− or I+ faintly violet	90–110(–140) × 30–45 μm	muriform, peripheral = central	norstictic acid	Singh and Singh (2017)	
79	*Diorygma sticticum* Sutjaritturakan, Kalb & Lücking	1–3 × 0.15–0.3 mm, 0.1 mm high	disc concealed to partly exposed			8, I+ strongly violet-blue	10–13 × 6–8 μm	submuriform with 3 transverse and 0–1 longitudinal septum, with thickened septa and rounded lumina	stictic, hypostictic, and cryptostictic acids	Lumbsch et al. (2011)	
80	*Diorygma streimannii* A.W. Archer & Elix	disciform, sessile, usually circular, sometimes distorted, 0.7–1.2 mm		exciple non–carbonized.	I–	1, I+ blue	120-160 × 32-40 μm	muriform	neotricone (major), norstictic acid (minor), salazinic acid (minor), norperistictic acid (minor) and protocetraric acid (minor)	Archer and Elix (2017)	
81	*Diorygma subalbatum* (Patw. & Makhija) B.O. Sharma & Makhija	straight to curved to flexuous, branched, 0.5–2.5 × 0.3–0.6 mm	disc narrow to broad	exciple convergent to divergent, non-carbonized, indistinctly striate	I+ blue violet	1–8, I+ violet	75–145 × 24–34 μm	muriform, peripheral = central	norstictic, and stictic acids present	Sharma and Makhija (2009b)	
82	*Diorygma subpruinosum* J. Sutjaritturakan & K. Kalb	ascomata numerous, short to long, ± flexuous and 1-branched, 0.4–3 × 0.4–1.2 mm	disc widely opened		I+weakly violet	1–2, without a gelatinous sheath, I+ violet	80–90 × 25–30 µm	richly muriform, peripheral = central, 16–17/4–5 locular	protocetraric acid (major), hypostictic acid (submajor) and mythylstictic acid (minor)	Sutjaritturakan et al. (2014)	
83	*Diorygma talisensis* (A.W. Archer) A.W. Archer	scattered, curved or sinuous, 1.5–3 × 0.2–0.4 mm	lips closed, sometimes becoming open	proper exciple uncarbonised		1, I+ blue	(90–)100–136 × 30–44 μm	muriform	stictic acid	Archer (2003, 2007)	= *Graphina talisensis* A.W. Archer
84	*Diorygma thailandicum* J. Sutjaritturakan & K. Kalb	numerous, distinctly flat, oval to oblong, ± flexuous, simple (with a few branches), 0.5–3.0 × 0.4–1.0 mm	disc widely open	exciple divergent,dark brown to carbonised	I+ violet	1, I+ weakly blue	87–105 × 27–32 µm	richly muriform, peripheral = central, 17–19/4–5 locular	protocetraric acid (major), stictic acid (major), cryptostictic acid (minor) and constictic acid (minor)	Sutjaritturakan et al. (2014)	
85	*Diorygma tiantaiense* Z.F. Jia	0.5–3 × 0.4–2 mm	disc open	sometimes sparse; exciple divergent, later	I–	1, with thin halo, I–	120–210 × 35–60 µm	muriform, peripheral = central	K+ red, P+ yellow to red; norstictic acid	Cui et al. (2024)	
86	*Diorygma tibellii* Kalb, Staiger & Elix	ascocarps often numerous, long, fexuous and branched, 0.5–5 × 0.3–0.6 mm	disc open	exciple divergent, totally uncarbonised, poorly developed	I– or laterally I+ weakly violet	(l)2–4, often with thick spore wall (1–3 μm), at least when young, I+ violet, wall thickening I–	55–90 × (16–)20–30 µm	muriform,15–20/4–8 locular	stictic acid (major), peristictic, substictic, constictic and cryptostictic acids (in various amounts), sometimes faint traces of hypostictic acid. Salazinic, consalazinic, 3–0 methylcon-salazinic and norstictic acids	Kalb et al. (2004)	pycnidia immersed in small thalline warts with a brownish ostiolum; conidia hyaline, bacilliform, 3–4 × 1 μm.
87	*Diorygma tinctorium* Eschw.	apothecia oblong and linearly elongate, somewhat branched, enclosed within a thallus that eventually	splits open, disc is flat and channeled (reddish in color in some cases)			thecae large, ovate-cylindrical, with multiple annulations				Eschweiler (1824)	
88	*Diorygma tocantinsense* S.C. Feuerst., I.P.R. Cunha & Aptroot	0.5–6.0 × 1.0 mm, margins entire, not corticate	disc narrowly exposed		I–	8, hyaline, broadly fusiform with rounded ends, surrounded by c. 5 mm wide gelatinous sheath, I+ faintly blue	24–40 × 10–15 µm	muriform	thallus K+ deep yellow, P+orange. TLC: protocetraric acid	Feuerstein et al. (2014)	
89	*Diorygma toensbergianum* Aptroot & M. Cáceres,	ascomata not observed				not observed			thallus UV+ yellow, K+ yellow > red, P–. TLC: lichexanthone and norstictic acid	Aptroot et al. (2023)	characterized by the presence of lichexanthone and norstictic acid
90	*Diorygma tuberculosum* (Stirt.) Kalb, Staiger & Elix	roundish, oval to oblong, ± fexuous and branched,1–5 × 0.4–1 mm; bulging	discs first narrow, later on wide	exciple divergent, uncarbonised, poorly developed	I+ weakly bluish violet	1, I- or only weakly I+ violet.	100–130 × 44–48 μm	muriform, peripheral < central	norstictic and connorstictic acids	Kalb et al. (2004)	
91	*Diorygma verrucirimosum* B.O. Sharma & Makhija	1–2 mm long, ends acute to obtuse	disc narrow to wide, sunken, 0.2–0.4 mm wide	exciple covered by crystals, divergent, uncarbonized, base distinctly orange brown	I+ blue	1, I+ blue	97–126 × 25–33 μm	muriform, peripheral = central	norstictic and salazinic acids present	Sharma and Makhija (2009a)	
92	*Diorygma weii* Wei Wu & S.B. Fu, sp. nov.	prominent, stellately branched, curved, and either terminally rounded or acute, 2–7 mm long, and 0.1–0.4 mm wide	disc closed to slit-like, covered with a thin white pruina	exciple uncarbonized, brown at apex, pale yellowish brown towards base	I+ violet	1, I+ violet	(55–)73–119(–142) × (11–)18–35(–41) μm	muriform, 24–28/6–9 locular, peripheral = central	thallus K+ reddish brown, C–, KC+ yellow, P+ yellow, TLC: norstictic acid	this study	
93	*Diorygma wallamanensis* A.W. Archer & Elix	straight, curved or sinuous, branched, 1–4(–5) × 0.2–0.3 mm wide	lips initially closed, opening to reveal a narrow, reddish-brown epruinose	exciple non-carbonized, indistinct	I–	8 per ascus, 2-seriate, I+ blue-violet	40–50 × 8–10 μm	transversely 10–12-locular	stictic acid (major) and peristictic acid (minor)	Archer and Elix (2008)	
94	*Diorygma wilsonianum* (Müll. Arg.) A.W. Archer	perithecium dark brown-black, very thin, lacking a basal structure	disc flat, widely open, black			8, fusiform	45–50 × 8–9μm	10–16-loculate		Archer (2005)	= *Graphis wilsonianum* Müll. Arg.

During a field survey of lichens in Guizhou Province, we discovered several specimens that formed phylogenetically distinct clades within *Diorygma*. Following detailed morphological and chemical analyses and comparison with all known species, we propose these as four new species.

## ﻿Material and methods

### ﻿Sample collection and morphological observations

Specimens were collected from the Leigong Shan National Nature Reserve in Leishan County and the Yueliangshan Nature Reserve in Congjiang County, Guizhou Province, China. All voucher specimens are deposited in the Lichen Herbarium of the Kunming Institute of Botany (KUN-L), Chinese Academy of Sciences, Yunnan, China. External morphological characteristics of the thallus and ascomata were examined using a stereomicroscope (OLYMPUS SZX16, Japan) and photographed with a fitted digital camera (AOR B32, China). Anatomical features, including the exciple, hymenium, paraphyses, asci, and ascospores, were observed using a light microscope (OLYMPUS BX53, Japan) based on hand-cut longitudinal sections of apothecia manually prepared with a razor blade. The sections were immersed in distilled water, and images were captured with a digital camera (OLYMPUS DP72, Japan). Lugol’s iodine solution (I) was used to stain and examine the hymenium. Photographic plates were assembled using Adobe Photoshop CC 2019 (Adobe Systems, USA). Measurements were conducted using ImageJ software (v. 1.50d) and are presented as (min–) (x̄ – SD) – (x̄ + SD) (–max), where x̄ is the arithmetic mean and SD is the standard deviation (rounded to the nearest 0.5 µm), followed by the number of observations (n) when n ≥ 10. The terms ‘min’ and ‘max’ represent the extreme values observed (Zhurbenko and Diederich 2024).

### ﻿Chemical component analysis

The color reactions of the thallus and medulla were tested using the following reagents: 10% potassium hydroxide (KOH, K), saturated sodium hypochlorite solution (NaClO, C), and a saturated solution of *p*-phenylenediamine in 95% ethanol (P). Lichen substances were analyzed by thin-layer chromatography (TLC) using the solvent C system (formic acid/acetic acid, v/v = 200/30) (Culberson 1972).

### ﻿DNA extraction, PCR amplification, and sequencing

Genomic DNA was directly extracted from apothecia using a fungal genomic DNA extraction kit (Solarbio, China), following the manufacturer’s protocol. Mitochondrial small subunit rRNA (mtSSU) sequences were amplified using the primer pairs mrSSU1/mrSSU3R, while large subunit ribosomal DNA (LSU) sequences were amplified using the primer pairs LR5/LROR and AL2R/LR6 (Vilgalys and Hester 1990; Zoller et al. 1999; Mangold et al. 2008; Kraichak et al. 2015). Polymerase chain reaction (PCR) was performed using a Bio-RAD T-100 thermal cycler in 25 μL reaction volumes, consisting of 12.5 μL of 2× PCR Mix (including dNTPs mix, Solarbio, China), 8 μL of double-distilled water (ddH_2_O), 1.0 μL of each 10 mM primer, and 2.5 μL of DNA template. The PCR conditions were as follows: an initial denaturation at 95 °C for 5 minutes, followed by 38 cycles of denaturation at 94 °C for 45 seconds, annealing at 50 °C for 60 seconds (for mtSSU) or 55 °C for 60 seconds (for LSU), and extension at 72 °C for 90 seconds, with a final extension at 72 °C for 10 minutes and held at 12 °C (Kraichak et al. 2015). The PCR products were visualized by 1% agarose gel electrophoresis and subsequently sequenced by Beijing Tsingke Biotech Co., Ltd. (Chongqing, China).

### ﻿Phylogenetic analyses

The sequencing results were evaluated by analyzing chromatograms using BioEdit Sequence Alignment software (version 7.0.9.0). Forward and reverse sequences were assembled using ContigExpress software (version 6.0.620.0). Preliminary taxonomic affiliation and potential sample contamination were confirmed by BLASTn searches on the NCBI website (https://blast.ncbi.nlm.nih.gov/Blast.cgi). Newly generated sequences were deposited in GenBank (Table 1). Additional sequences used for ingroup analysis were retrieved from the NCBI website (https://www.ncbi.nlm.nih.gov), and *Glyphis cicatricosa* was selected as the outgroup (Ansil et al. 2023). Phylogenetic analyses were conducted using the OFPT program (Zeng et al. 2023), following its protocol: each gene region dataset was initially aligned using the ‘auto’ strategy (based on data size) in MAFFT (Katoh and Standley 2013) and subsequently trimmed using the ‘gappyout’ method (based on gap distribution) in TrimAl (Capella-Gutiérrez et al. 2009). Single-gene phylogenetic trees were constructed using the IQ-TREE Web Server (http://iqtree.cibiv.univie.ac.at/) to confirm well-supported branches. The best-fit nucleotide substitution models for each dataset were selected based on the Bayesian Information Criterion (BIC) from 22 common DNA substitution models with rate heterogeneity, using ModelFinder (Kalyaanamoorthy et al. 2017). All datasets were concatenated with partition information for subsequent phylogenetic analyses. Maximum likelihood (ML) analysis was performed using ultrafast bootstrap approximation (Hoang et al. 2018), combined with the SH-like approximate likelihood ratio test (SH-aLRT) (Guindon et al. 2010) in IQ-TREE (Nguyen et al. 2015). The consensus tree was summarized based on the extended majority rule. Additionally, ML analysis was conducted using RAxML-HPC2 on ACCESS (version 8.2.12) in the CIPRES Science Gateway (https://www.phylo.org/portal2/login!input.action) with the GTRGAMMA model and a rapid bootstrap analysis of 1000 replicates (Miller et al. 2010; Stamatakis 2014). Bayesian inference was performed using MrBayes (Ronquist et al. 2012), with two parallel Metropolis-coupled Markov Chain Monte Carlo runs (one ‘cold’ chain and three heated chains), sampling trees every 1000 generations. The run was automatically terminated when the average standard deviation of split frequencies dropped below 0.01, and the resulting tree was summarized after discarding the first 25% of samples as burn-in. The resulting trees were visualized in FigTree v1.4.4 and further edited in Adobe Illustrator CC 2019. The new taxon was registered in Index Fungorum (2025) and Faces of Fungi (Jayasiri et al. 2015).

## ﻿Result

### ﻿Phylogenetic analyses

**Figure 1.**
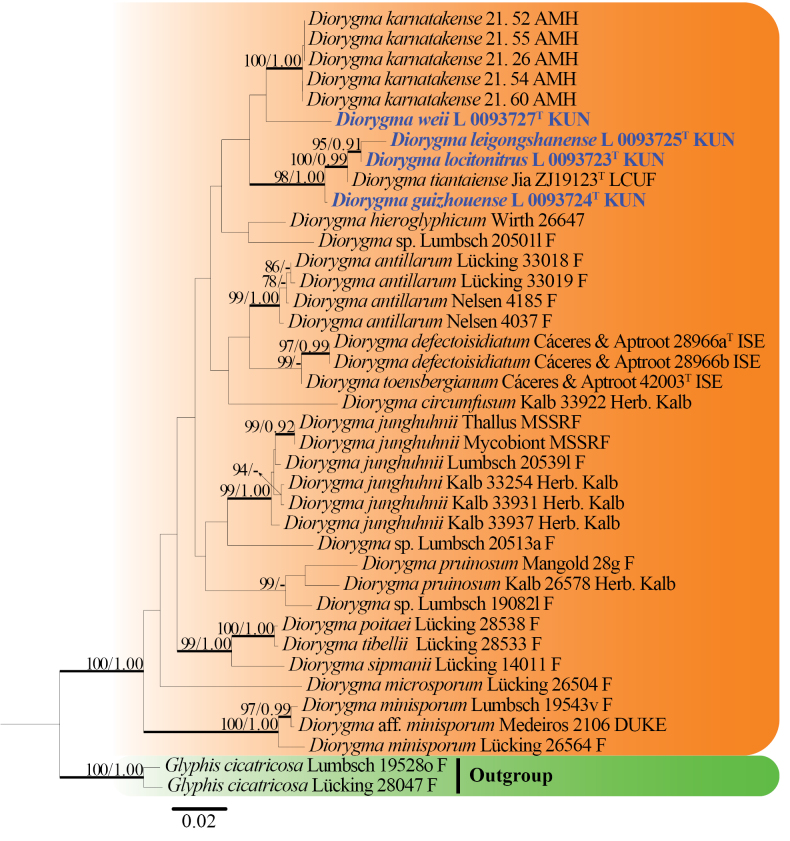
RAxML analysis based on combined LSU and mtSSU sequence data. Bootstrap support values for maximum likelihood (ML ≥ 75%) and Bayesian posterior probabilities (PP ≥ 0.90) are shown near the nodes as ML/PP. *Glyphis cicatricosa* (Lücking 28047 F and Lumbsch 19528o F) is used as the outgroup taxon. Newly generated sequences are shown in bold. Type strains are indicated as ^T^.

The final dataset comprised 22 taxa and 39 strains/vouchers, with 1562 aligned characters including gaps (LSU: 760 bp; mtSSU: 802 bp). The RAxML tree was constructed with a final ML optimization likelihood value of –6039.173664. The parameters for the GTR+I+G model of combined LSU and mtSSU were as follows: estimated base frequencies–A = 0.29, C = 0.19, G = 0.27, T = 0.25; substitution rates–AC = 0.66, AG = 2.55, AT = 1.69, CG = 0.76, CT = 7.83, and GT = 1.00. Bayesian posterior probabilities from MCMC analysis showed a final average standard deviation of split frequencies of 0.009999. The topologies from both ML and Bayesian analyses were verified manually and largely concurred (Fig. 1).

Fourteen known species of *Diorygma* formed a well-supported clade in the phylogenetic tree. Three new species–*D. leigongshanense*, *D. locitonitrus*, and *D. guizhouense*–clustered in a clade with *D. tiantaiense* Z.F. Jia, with strong support. In contrast, *Diorygma weii* was related to *D. karnatakense*, but this relationship received low support.

### ﻿Taxonomy

#### ﻿ Diorygma guizhouense

Taxon classificationFungiOstropalesGraphidaceae

Wei Wu & S.B. Fu sp. nov.

A1725E2A-C296-55EB-95E5-03FEC45D00F0

Index Fungorum: IF903744

Facesoffungi Number: FoF17083

Fig. 2

##### Etymology.

The specific epithet “*guizhouense*” refers to the location where the holotype was collected.

##### Holotype.

KUN-L 0093724

##### Description.

***Sexual morph: Thallus*** corticolous, crustose, thin, tightly attached to the substratum, pale grey to greenish grey, rough, dull, lacking isidia and soredia, prothallus absent. ***Apothecia*** lirelliform, scattered or aggregated, erumpent, simple or irregularly branched, curved, and either terminally rounded or acute, measuring 2–5 mm long and 0.2–0.4 mm wide. ***Disc*** narrow to slightly open, covered with a white pruina. ***Exciple*** uncarbonized, brown at apex, pale yellowish brown towards base. ***Epihymenium*** brown, 10–30 µm high. ***Hymenium*** hyaline, not inspersed, 150–210 μm high, I+ weakly blue-violet. ***Paraphysis*** anastomosing, filiform, 1–2.5 µm wide. *Hypothecium* weakly yellowish brown, 15–45 µm high. ***Asci*** fusiform, 118–222 × 37–82 μm, I–. ***Ascospores*** 1/ascus, hyaline, richly muriform, peripheral and central spore locules of ± equal size, ends with gelatinous caps, 24–32 × 5–12 locular, (104–)125–119(–214) × (30–)36–58(–77) μm (x̄ = 152 × 47 μm, n = 20), I–. ***Asexual moprh***: not observed.

##### Chemistry.

Thallus K+ reddish brown, C–, KC+ orange, P+ yellow, TLC: stictic acid, salazinic acid, norstictic acid.

##### Material examined.

China, • Guizhou Province, Congjiang County, Yueliangshan Nature Reserve, 25°20'8.56"N, 108°36'19.23"E, 987 m elev., 24 Oct. 2023, Ze Yang & Bo Liu, Y400 (holotype KUN-L 0093724).

##### Notes.

The new species *Diorygma guizhouense* is characterized by lirelliform apothecia, which are erumpent with a narrow to slightly open disc covered by a white pruina. The exciple is uncarbonized, the hymenium is hyaline, not inspersed, and reacts I+ blue-violet. The ascospores, one per ascus, are hyaline, richly muriform, with peripheral and central spore locules of approximately equal size, containing 24–32 × 5–12 locular, measuring 125–179 × 36–58 μm, I–. Chemically, this species contains stictic, salazinic, and norstictic acids.

**Figure 2.**
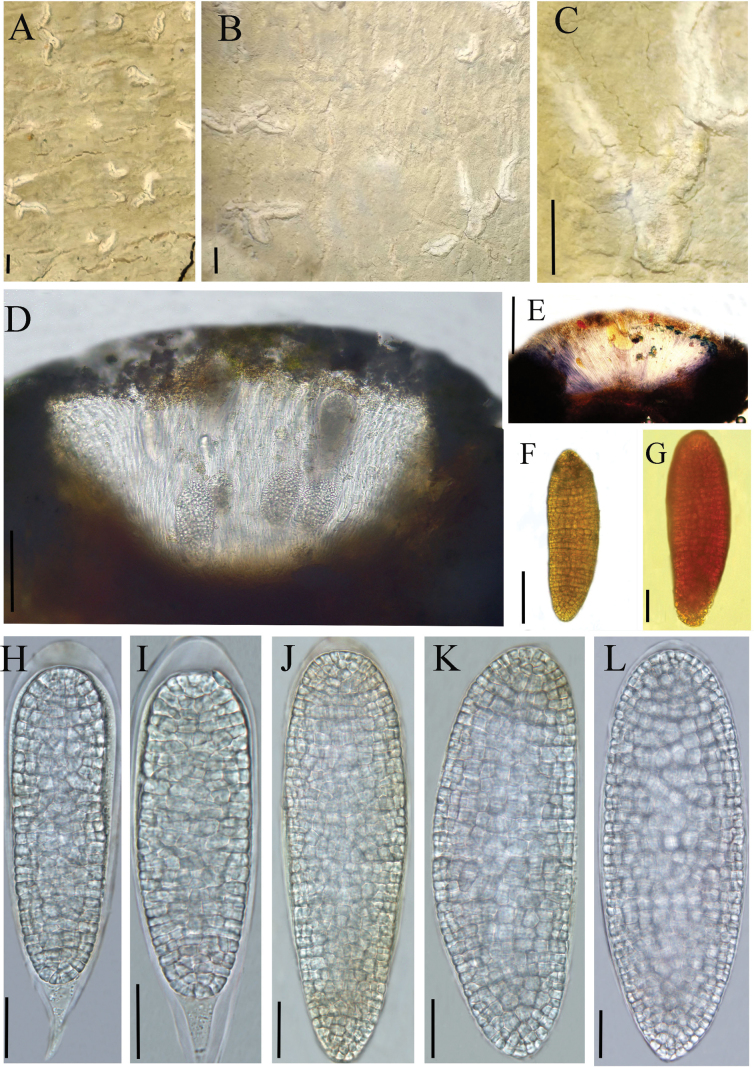
*Diorygma guizhouense* (KUN-L 0093724, holotype). A–C. Thallus with ascomata; D. Cross section of apothecium; E. Cross section of apothecium (in IKI); H, I. Asci; J–L. Ascospores (in water); F, G. Ascospore (in IKI). Scale bars: 1 mm (A–C); 100 µm (D); 200 µm (E); 50 µm (F); 20 µm (G, H–L).

Phylogenetic analysis based on combined LSU and mtSSU sequence data indicates that *D. guizhouense* is closely related to *Diorygma tiantaiense* (Fig. 1). A comparison with *D. tiantaiense* reveals a 0.97% nucleotide difference in the LSU region (7/723 bp). Morphologically, *D. guizhouense* differs by having a narrowly open disc (vs. fully open) and chemically by having a hymenium that reacts I+ blue-violet (vs. I–). According to the TLC result, the new species contains stictic and salazinic acids rather than only norstictic acid in *D. tiantaiense* (Cui et al. 2024).

According to the taxonomic key provided by Feuerstein et al. (2014), *D. guizhouense* is morphologically similar to *D. dandeliense*, which was later synonymized to *D. karnatakense* (Ansil et al. 2023). However, *D. guizhouense* and *D. karnatakense* occupy distinct clades in the phylogenetic tree, supporting their separation at the species level. Chemotaxonomically, TLC analysis shows that *D. guizhouense* contains stictic, salazinic, and norstictic acids, whereas *D. karnatakense* lacks stictic acid, and the ascospores of *D. guizhouense* exhibit a negative iodine reaction (I–), in contrast to the I+ violet reaction reported in related species (Ansil et al. 2023). The nucleotide comparison reveals clear differences between the two species: 4.28% (31/725 bp) for LSU and 3.45% (25/725 bp) for mtSSU.

#### ﻿ Diorygma leigongshanense

Taxon classificationFungiOstropalesGraphidaceae

Wei Wu & S.B. Fu sp. nov.

542CF60F-DFD2-5BF4-BE3E-1E9704B87EA7

Index Fungorum: IF903745

Facesoffungi Number: FoF17549

Fig. 3

##### Etymology.

The specific epithet “*leigongshanense*” refers to the location where the holotype was collected.

##### Holotype.

KUN-L 0093725

##### Description.

***Sexual morph: Thallus*** corticolous, crustose, thin, tightly attached to the substratum, pale grey to greenish grey, rough, dull, lacking isidia and soredia, prothallus absent. ***Ascomata*** lirellate, numerous, oblong to long, ± flexuous, simple or with a few branches, measuring 0.7–2.5 mm long and 0.4–1.2 mm wide. ***Disc*** surrounded by entire raised thalline margins, widely open, covered with a thin, pale yellowish pruina. ***Exciple*** uncarbonized, basally and laterally brownish. ***Epihymenium*** brown, 15–41 µm high. ***Hymenium*** hyaline, not inspersed, 160–350 μm high, I–. ***Paraphysis*** anastomosing, filiform, 1–2.5 µm wide. ***Hypothecium*** brown, 20–48 µm high. ***Asci*** fusiform, 106–202 × 28–58 μm, I–. ***Ascospores*** 1/ascus, hyaline, richly muriform, peripheral and central spore locules of ± equal size, 20–42 × 5–10 locular, (95–)119–170(–194) × (22–)27–46(–54) μm (x̄ = 144 × 37 μm, n = 20), I–. ***Asexual moprh***: not observed.

##### Chemistry.

Thallus K+ reddish brown, C–, KC+ yellow, P+ yellow, TLC: stictic acid, salazinic acid, norstictic acid.

##### Material examined.

China, • Guizhou Province, Leishan County, Leigong Mountain National Nature Reserve, 26°22'43.16"N, 108°11'42.65"E, 1681 m elev., on bark, 27 Oct. 2023, Ze Yang & Bo Liu, LGS207 (holotype KUN-L 0093725).

##### Notes.

This species is characterized by its erumpent lirelliform apothecia, with discs surrounded by entire, raised thalline margins that are widely open and covered with a thin, pale yellowish pruina. The exciple is uncarbonized, and the hymenium is hyaline, non-inspersed, and I–. Spores are single per ascus, hyaline, richly muriform, with peripheral and central spore locules of approximately equal size, 20–42 × 5–10 locular, measuring 119–170 × 27–46 μm. Chemically, this species contains stictic, salazinic, and norstictic acids.

**Figure 3.**
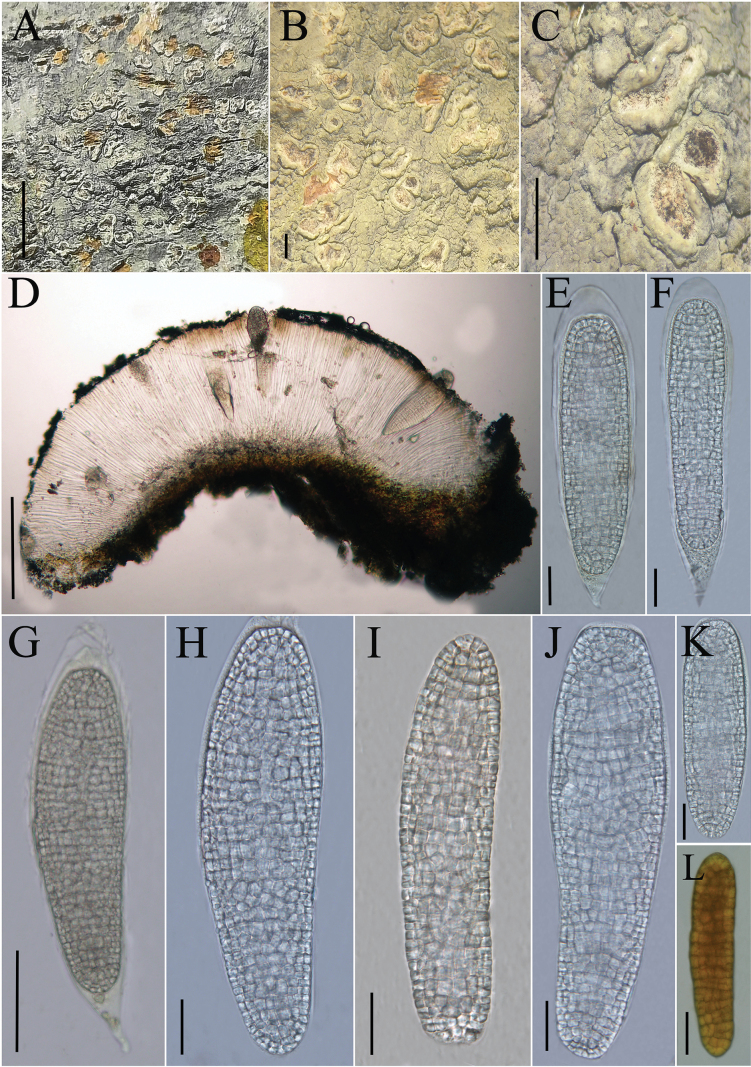
*Diorygma leigongshanense* (KUN-L 0093725, holotype). A–C. Thallus with ascomata; D. Cross section of apothecium; E–G. Ascus; H–K. Ascospores (in water); L. Ascospore (in IKI). Scale bars: 1 cm (A); 1 mm (B, C); 200 µm (D); 20 µm (E, F); 50 µm (G); 20 µm (H–L).

Phylogenetic analysis based on combined LSU and mtSSU sequence data places *D. leigongshanense* as closely related to *Diorygma tiantaiense* (Fig. 1). However, *Diorygma leigongshanense* differs chemically by the presence of stictic and salazinic acids (vs. only containing norstictic acid). Morphologically, *D. leigongshanense* has lirelliform apothecia, in contrast to the oval, open, and raised ascocarps of *D. tiantaiense* (Cui et al. 2024).

*Diorygma leigongshanense* is morphologically similar to *D. rufopruinosum* (A.W. Archer) Kalb, Staiger & Elix. However, it can be distinguished by its ascospore septation pattern: in *D. leigongshanense*, the peripheral cells and central cells are of similar size, whereas in *D. rufopruinosum*, the peripheral cells are noticeably smaller. *Diorygma leigongshanense* contains stictic, salazinic, and norstictic acids, while *D. rufopruinosum* produces protocetraric acid and lacks stictic acid (Kalb et al. 2004).

Morphologically, *Diorygma leigongshanense* also shares similarities with *D. chumphonense* Sutjaritturakan & K. Kalb, but it can be distinguished by having longer ascospores (119–170 μm vs. 95–110 μm), peripheral cells of equal size to the central ones (vs. peripheral cells distinctly smaller), lacking stictic acid (vs. presence), and the I– (vs. I+ blue-violet) (Sutjaritturakan et al. 2014).

#### ﻿ Diorygma locitonitrus

Taxon classificationFungiOstropalesGraphidaceae

Wei Wu & S.B. Fu sp. nov.

3CB8A1A7-4AF9-51E5-AB81-69A4AEEF6973

Index Fungorum: IF903746

Facesoffungi Number: FoF17550

Fig. 4

##### Etymology.

“locitonitius” combines “loci,” signifying locality, with “tonitius,” the Latin word for thunder, to mean “of the locality of thunder,” denoting the location where the holotype was found.

##### Holotype.

KUN-L 0093723

##### Description.

***Sexual morph: Thallus*** corticolous, crustose, thin, tightly attached to the substratum, pale grey to greenish grey, rough, dull, lacking isidia and soredia, prothallus absent. ***Apothecia*** lirelliform, scattered or aggregated, erumpent, simple or irregularly branched, curved, and either terminally rounded or acute, measuring 0.5–2.5 mm long and 0.2–0.6 mm wide. ***Disc*** narrow to open, covered with a white pruina. ***Exciple*** uncarbonized, brown at apex, pale yellowish brown towards base. ***Epihymenium*** brown, 10–42 µm high. ***Hymenium*** hyaline, not inspersed, 180–350 μm high, I+ weakly blue-violet. ***Paraphysis*** anastomosing, filiform, 1–2.5 µm wide. ***Asci*** fusiform, 112–260 × 30–81 μm, I–. ***Ascospores*** 1/ascus, hyaline, richly muriform, peripheral cells distinctly smaller than central ones, 26–40/6–15 locular, (105–)117–189(–247) × (25–)33–60(–76) μm (x̄ = 153 × 47 μm, n = 20), I–. ***Asexual moprh***: not observed.

##### Chemistry.

Thallus K+ reddish brown, C–, KC+ orange, P+ yellow, TLC: constictic acid, salazinic acid, norstictic acid.

##### Material examined.

China, • Guizhou Province, Leishan County, Leigong Mountain National Nature Reserve, 26°22'53.94"N, 108°11'46.76"E, 1771 m elev., on bark, 27 Oct. 2023, Ze Yang & Bo Liu, LGS256-1 (holotype KUN-L 0093723).

##### Notes.

This species is characterized by its lirelliform apothecia, which are erumpent with narrow to open discs covered by a white pruina; the exciple is uncarbonized, and the hymenium is hyaline, not inspersed, and reacts I+ weakly blue-violet. The ascospores, one per ascus, are hyaline, richly muriform, with peripheral cells distinctly smaller than central ones, containing 26–40 × 6–15 locular, measuring 117–189 × 33–60 μm, I–. Chemically, this species contains constictic, salazinic, and norstictic acids.

**Figure 4.**
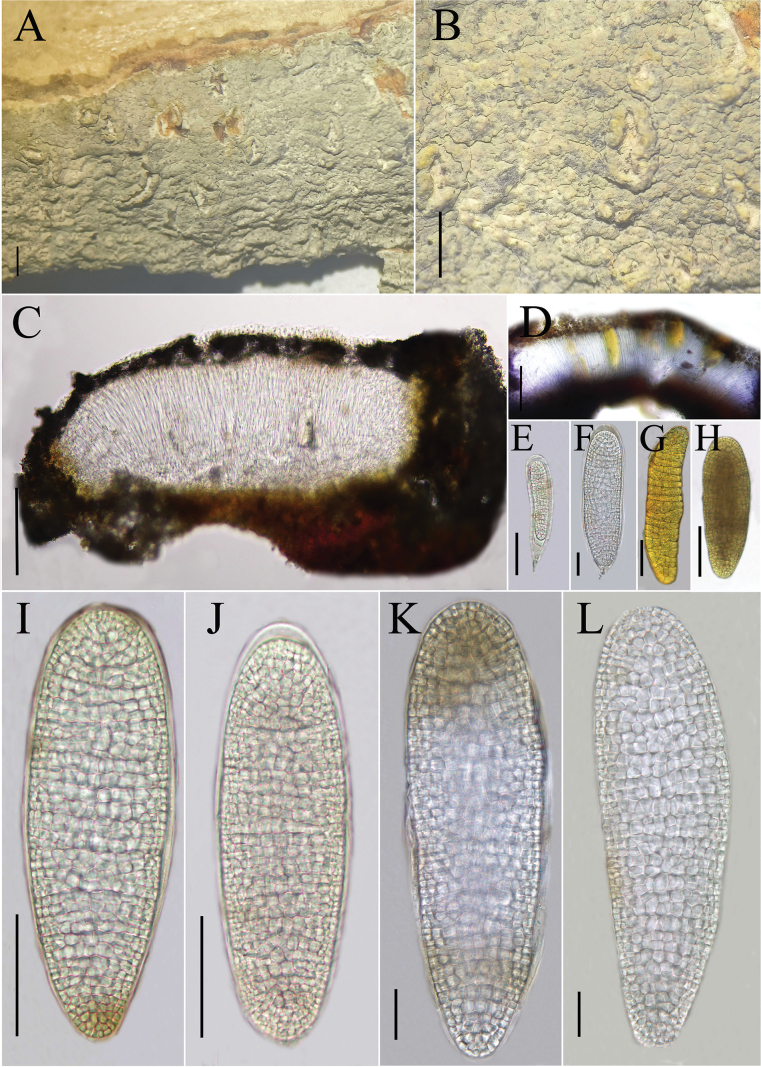
*Diorygma locitonitrus* (KUN-L 0093723, holotype). A, B. Thallus with ascomata; C. Cross section of apothecium; D. Cross section of apothecium (in IKI); E, F. Ascus; I–L. Ascospores (in water); G, H. Ascospore (in IKI). Scale bars: 1 mm (A, B); 100 µm (C, D); 50 µm (E, H, I, J); 20 µm (F, J–L).

Phylogenetic analysis based on combined LSU and mtSSU sequence data places *Diorygma locitonitrus* as closely related to *D. tiantaiense* (Fig. 1). However, nucleotide comparison of the LSU reveals a difference between *D. locitonitrus* of 0.80% (6/758 bp) between the two species. The new taxon is chemically distinct from the presence of both norstictic and salazinic acids, while *D. tiantaiense* contains only norstictic acid. In addition, the hymenium of *D. locitonitrus* exhibits a weakly I+ blue-violet reaction in Lugol’s solution, in contrast to the I– reaction in *D. tiantaiense* (Cui et al. 2024).

*Diorygma locitonitrus* is morphologically similar to *D. chumphonense* but differs in the larger ascospores (117–189 × 33–60 μm vs. 95–110 × 37–40 μm) and the iodine reaction of the hymenium (I–, vs. I+ weakly violet). Additionally, the ascospores of *D. locitonitrus* are I–, in contrast to the I+ violet reaction observed in *D. chumphonense* (Sutjaritturakan et al. 2014).

#### ﻿ Diorygma weii

Taxon classificationFungiOstropalesGraphidaceae

Wei Wu & S.B. Fu sp. nov.

BFC6EFA1-FFA3-56AF-BDF5-8400FEE7E600

Index Fungorum: IF903747

Facesoffungi Number: FoF17617

Fig. 5

##### Etymology.

The species epithet “*weii*” honors Professor Jiangchun Wei (Chinese Academy of Sciences), a venerable lichenologist, for his pioneering contributions to lichenology in China.

##### Holotype.

KUN-L 0093727

##### Description.

***Sexual morph: Thallus*** corticolous, crustose, thin, tightly attached to the substratum, milky white, with a slight greenish tint, rough, dull, lacking isidia and soredia, prothallus absent. ***Apothecia*** lirelliform, prominent, stellately branched, curved, and either terminally rounded or acute, measuring 2–7 mm long and 0.1–0.4 mm wide. ***Disc*** closed to slit-like, covered with a thin white pruina. Proper margin conspicuous. ***Exciple*** uncarbonized, brown at apex, pale yellowish brown towards base. ***Hymenium*** hyaline, not inspersed, 150–210 μm high, I+ violet. ***Paraphysis*** anastomosing, filiform, 1–2 µm wide. ***Asci*** fusiform, 60–155 × 18–45 μm, I– or I+ violet. ***Ascospores*** 1/ascus, hyaline, richly muriform, peripheral and central spore locules of ± equal size, ends with gelatinous caps, 24–28/6–9 locular, (55–)73–119(–142) × (11–)18–35(–41) μm (x̄ = 96 × 27 μm, n = 20), I+ violet. ***Asexual moprh***: not observed.

##### Chemistry.

Thallus K+ reddish brown, C–, KC+ yellow, P+ yellow, TLC: Norstictic acid.

##### Material examined.

China, • Guizhou Province, Leishan County, Leigong Mountain National Nature Reserve, 26°20'33.94"N, 108°17'23.94"E, 831 m elev., on the bark, 25 Oct. 2023, Ze Yang & Bo Liu, Coll. No. LGS57 (holotype KUN-L 0093727).

##### Notes.

This new species is characterized by its lirelliform apothecia, which are prominent with a closed-to-slit-like disc covered by a thin white pruina. The exciple is uncarbonized, and the hymenium is hyaline, not inspersed, and reacts I+ violet. Spores are single per ascus, hyaline, richly muriform, with peripheral and central spore locules of approximately equal size, 24–28 × 6–9 locular, measuring 73–119 × 18–35 μm, I+ violet. Chemically, this species contains only norstictic acid.

**Figure 5.**
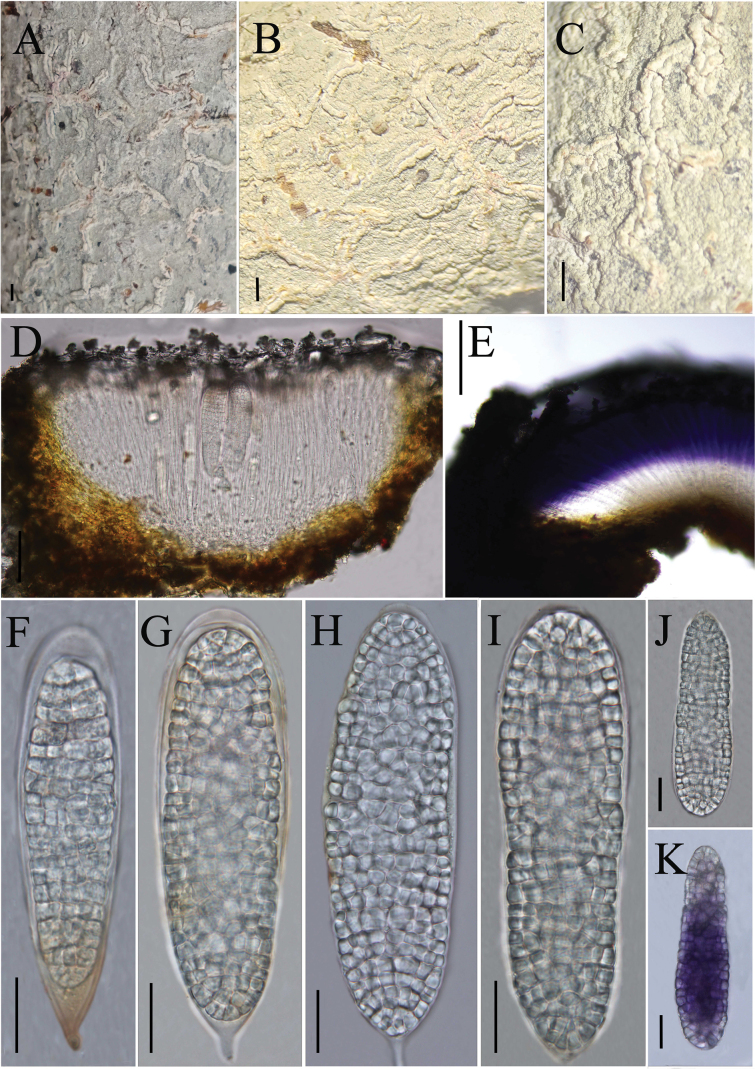
*Diorygma weii* (KUN-L 0093727, holotype). A–C. Thallus with ascomata; D. Cross section of apothecium; E. Cross section of apothecium (in IKI); F–H. Ascus; I, J. Ascospores (in water); K. Ascospore (in IKI). Scale bars: 1 mm (A–C); 50 µm (D); 100 µm (E); 20 µm (F–K).

Phylogenetic analysis based on the combined data of LSU and mtSSU sequences indicates that *D. weii* forms a clade with *D. karnatakense* (Fig. 1). However, nucleotide divergence in mtSSU between the two species is 3.52% (23/653 bp). Morphologically, *D. weii* has asci containing a single spore, versus 1–8-spored asci in *D. karnatakense*. Chemically, *D. weii* only produces norstictic acid, whereas *D. karnatakense* contains both norstictic and salazinic acids (Ansil et al. 2023).

*Diorygma weii* shares morphological similarities with *Diorygma inaequale* and *D. dealbatum* B.O. Sharma & Makhija. However, both *D. inaequale* and *D. dealbatum* contain both salazinic and norstictic acids, whereas the new species produces only norstictic acid (Sharma and Makhija 2009a).

## ﻿Discussion

Morphological features and chemical compounds are generally used in the classification of *Diorygma* species (Kalb et al. 2004). Chemically, most species exhibit remarkable diversity in secondary metabolites–such as norstictic acid, stictic acid, cryptostictic acid, and protocetraric acid–which serve as key taxonomic markers. Typically, a single species produces one or several of these characteristic metabolites (Kalb et al. 2004). With the rapid advancement of molecular technologies, phylogenetic analyses have become an indispensable tool in species identification and evolutionary studies. Molecular data provide objective genetic evidence that enables the precise differentiation of morphologically similar or cryptic species, help clarify taxonomic uncertainties including synonymies, and facilitate the discovery of new taxa. Phenotypic characteristics alone are sometimes insufficient for resolving taxonomic ambiguities due to environmental influences and developmental stage. In contrast, genetic data are relatively stable. DNA sequences from LSU and mtSSU are usually considered reliable molecular markers, and phylogenetic trees based on these genes can confirm the monophyletic nature of species defined by morphology and correct misclassifications caused by overlapping features of sporangia or ascospores within *Diorygma* (Cui et al. 2024; Aptroot et al. 2023). Therefore, this study adopts an integrative taxonomic approach, combining phylogenetic analyses, morphological characteristics, and chemical profiling to achieve a comprehensive and accurate classification of new *Diorygma* species.

Most *Diorygma* species have stable phylogenetic positions with stronger statistical support in multigene datasets than in single-gene analyses (Ansil et al. 2023). The phylogenetic tree based on LSU + mtSSU sequence data provided high-resolution species delimitation within *Diorygma*. *Diorygma guizhouense*, *D. leigongshanense*, and *D. locitonitrus* formed a highly supported clade (ML/PP ≥ 90/0.90) with *D. tiantaiense*. Although *D. weii* and *D. karnatakense* formed a distinct clade, this relationship received weak support (ML/PP < 70/0.90). All four proposed species occupy distinct phylogenetic positions.

Approximately 90 *Diorygma* species are currently recognized and categorized by ascospore number into two morphological groups (Table 2). Among the 38 monosporate species, our four new taxa all belong to this group. Thirteen monosporate species produce salazinic acid–a feature shared by *D. guizhouense* and *D. leigongshanense*. These two species are further distinguished by co-occurring salazinic and stictic acids, a combination previously documented only in *D. angusticarpum* Sutjaritturakan & Kalb and *D. salazinicum* Sutjaritturakan & Kalb.

Morphological and chemical comparisons reveal that *D. guizhouense* can be distinguished from *D. angusticarpum* by its larger ascospores (125–179 × 36–58 μm vs. 90–110 × 30–37 μm) and the presence of norstictic acid (vs. cryptostictic acid) (Sutjaritturakan et al. 2014). Additionally, *D. guizhouense* differs from *D. salazinicum* in having a narrower disc (vs. widely opened discs), a weakly I+ blue-violet hymenium reaction (vs. I–), and the presence of norstictic acid (vs. cryptostictic acid) (Sutjaritturakan et al. 2014).

Comparative analysis reveals that *D. leigongshanense* exhibits unique diagnostic features when contrasted with similar species. Compared to *D. angusticarpum*, the new species displays markedly larger ascospores (119–170 × 27–46 μm vs. 90–110 × 30–37 μm), a distinctly broader disc (vs. slit-like), an I– hymenium (vs. I+ violet), and contains norstictic acid (vs. cryptostictic acid) (Sutjaritturakan et al. 2014). These chemical and morphological differences also distinguish *D. leigongshanense* from *D. salazinicum*, particularly in the iodine reaction patterns of the ascospores (I– vs. I+ violet) and secondary metabolite profiles (norstictic acid vs. cryptostictic acid) (Sutjaritturakan et al. 2014).

Taxonomic evaluation of ascospore septation (cell size) in monosporate *Diorygma* species revealed two distinct morphological types: those with peripheral cells smaller than central cells and those with cells of equal size. The newly described *D. locitonitrus* possesses the former pattern, a characteristic shared with nine other monosporate species. Notably, only three previously documented species–*D. reniforme* (Fée) Kalb, Staiger & Elix, *D. rufopruinosum*, and *D. salvadoriense* Kalb, Staiger & Elix̄exhibit both this septation pattern and the presence of salazinic acid. *Diorygma locitonitrus* can be distinguished from these chemically similar species by the absence of protocetraric acid (vs. present in *D. reniforme* and *D. rufopruinosum*) and the absence of pycnidia (vs. present in *D. salvadoriense*) (Kalb et al. 2004). These consistent differences in secondary metabolite profiles and reproductive structures provide robust criteria for delimiting *D. locitonitrus* from related taxa.

Among monosporate *Diorygma* species, seven taxa–*D. australasicum* (Elix) Lücking, Elix & A.W. Archer, *D. isabellinum* (Zahlbr.) Z.F. Jia & Lücking, *D. longilirellatum* B.O. Sharma & Makhija, *D. roseopruinatum* Papong, Lücking & Parnmen, *D. soozanum* (Zahlbr.) M. Nakan. & Kashiw., *D. spilotum* (Stirt.) Pushpi Singh & Kr.P. Singh, and *D. tiantaiense*–exclusively produce norstictic acid (Table 2), warranting detailed comparison with *D. weii*, which shares this chemical profile. *Diorygma weii* is distinguished by its unique thallus morphology (milky white with a slight greenish tint) and apothecial features (stellately branched with closed to slit-like discs). Key diagnostic characters that distinguish *D. weii* from related species include: (1) From *D. roseopruinatum* by thallus color (milky white vs. light grey), and disc exposure and pruinosity (closed, slit-like white pruinose vs. narrow brown with pink-red pruina) (Papong et al. 2014); (2) From *D. australasicum* by the absence of isidia (Archer and Elix 2009); (3) From *D. spilotum* by branching of lirellae (prominent stellately branched vs. simple immersed) and disc pruinosity (white pruinose vs. epruinose) (Singh and Singh 2017); (4) From *D. isabellinum* by branching of lirellae (stellately vs. single/rarely branched) and iodine reaction (I+ violet hymenium vs. I–) (Jia and Lücking 2017); (5) From *D. longilirellatum* by thallus color (milky white vs. greenish-grey) and disc exposure (closed vs. slightly open) (Sharma and Makhija 2009b); (6) From *D. tiantaiense* by smaller ascospores (73–119 × 18–35 μm vs. 120–210 × 35–60 μm), disc exposure (closed vs. open), and iodine reactions (I+ violet vs. I– for both hymenium and ascospores) (Cui et al. 2024); (7) From *D. soozanum* by narrower ascospores (73–119 × 18–35 μm vs. 110–140 × 35–45 μm) and disc exposure (closed vs. initially narrow then wide) (Kalb et al. 2004).

This study describes four new species of the lichen genus *Diorygma* from China. Each species exhibits distinctive morphological features and secondary metabolite profiles, confirmed through colorimetric tests and thin-layer chromatography. These findings contribute to a deeper understanding of *Diorygma* diversity in China and underscore the value of combining traditional taxonomy with molecular and chemotaxonomic approaches. A comprehensive species checklist is also provided to aid in the identification and comparison of known *Diorygma* taxa.

## Supplementary Material

XML Treatment for Diorygma guizhouense

XML Treatment for Diorygma leigongshanense

XML Treatment for Diorygma locitonitrus

XML Treatment for Diorygma weii

## ﻿Additional information

### Use of AI

No use of AI was reported.

### Author ORCIDs

Wei Wu https://orcid.org/0009-0009-1191-6667

Shu-Hao Jiang https://orcid.org/0009-0009-8619-2667

Lin-Shan Chai https://orcid.org/0009-0005-2458-0454

He-Yun Bo https://orcid.org/0009-0003-8641-1619

Ruvishika S. Jayawardena https://orcid.org/0000-0001-7702-4885

Shao-Bin Fu https://orcid.org/0000-0001-9932-1346

Qing-Feng Meng https://orcid.org/0000-0001-9814-8238

### Data availability

All of the data that support the findings of this study are available in the main text.
